# Using peer role-playing to improve students’ clinical skills for musculoskeletal physical examinations

**DOI:** 10.1186/s12909-021-02742-4

**Published:** 2021-06-05

**Authors:** Kazuyo Yamauchi, Yoko Hagiwara, Nahoko Iwakura, Saori Kubo, Azusa Sato, Tadahiko Ohtsuru, Ken Okazaki, Yumiko Okubo

**Affiliations:** 1 Department of Medical Education, Tokyo Women’s Medical University, 8-1, Kawada-cho, Shinjuku-ku, Tokyo, 162-8666 Japan; 2 Department of Orthopaedic Surgery, Tokyo Women’s Medical University, 8-1, Kawada-cho, Shinjuku-ku, Tokyo, 162-8666 Japan; 3 Department of Chemistry, Tokyo Women’s Medical University, 8-1, Kawada-cho, Shinjuku-ku, Tokyo, 162-8666 Japan; 4 Centre for Medical Education, Teikyo University School of Medicine, 2-11-1, Kaga, Itabashi-ku, Tokyo, 173-8605 Japan

**Keywords:** Musculoskeletal physical examination, Clinical reasoning and diagnosis, Peer role-playing simulation, Workplace-based assessment, Mini-CEX

## Abstract

**Background:**

The traditional curriculum for medical students in Japan does not include sufficient opportunities for students to develop their skills for musculoskeletal (MSK) examination and clinical reasoning and diagnosis. Therefore, an effective programme is required to help medical students and residents improve their clinical skills in MSK. This paper aims to assess the clinical skills of medical students who have participated in a peer role-playing simulation programme using a mini clinical evaluation exercise (mini-CEX).

**Methods:**

Participants were 90 female medical students who were completing their first orthopaedic clinical clerkship. They were divided into two groups. The simulation group participated in a role-play focussed on MSK cases as low-fidelity simulation, a structured debriefing with the course supervisor, and a self-reflection on Day 1 (*n* = 64). The control group did not participate in the role-play due to randomised clerkship schedules (*n* = 26). On Day 2 of the intervention, we observed and assessed all participants’ performances during MSK outpatient encounters using the mini-CEX. We compared the mini-CEX score between the simulation group and the control group; the Wilcoxon rank-sum test was used for statistical analysis.

**Results:**

The mini-CEX scores for physical examination, clinical reasoning and diagnosis, and overall clinical competency were significantly higher in the simulation group than in the control group (*p* < .05, physical examination: *p* = .014, clinical reasoning: *p* = .042, overall: *p* = .016). These findings suggest that medical students who partake in a peer role-playing simulation programme could experience improved clinical skills for physical examination, clinical reasoning and diagnosis, and overall clinical competency in real-life MSK outpatient encounters.

**Conclusions:**

Through a mini-CEX assessment, our findings indicate that medical students who participated in our peer role-playing simulation programme have improved clinical skills. Peer role-playing as a low-fidelity simulation and practical educational opportunity will enable educators to polish the competency of medical students in musculoskeletal physical examinations and clinical reasoning and diagnosis in a clinical setting.

## Background

As we enter the era of a super-aged society, we can expect an increase in the number of individuals who experience musculoskeletal (MSK) pain and impairment in joint and spine function. It is estimated that MSK and connective tissue diseases rank behind digestive and cardiovascular diseases in terms of outpatient visits, accounting for 12 % of all outpatient hospital consultations in Japan (Overview of the citizen life basic survey in 2016, Overview of Patient Survey in 2017). Considering this pattern, sound physical examination and clinical reasoning skills are essential for physicians to rapidly and accurately diagnose MSK conditions of the limbs and spine to practice in primary care. Currently, in Japan, the medical education curriculum provides few opportunities for learners to focus on physical examination. As such, there is a need to provide more educational opportunities for MSK physical examination and clinical reasoning in the medical curriculum. In the UK, the gait-arms-legs-spine (GALS) programme has been strategically included in undergraduate medical education to improve learners’ competence with MSK examination and diagnosis [1]. However, even this well-thought-out initiative was reported to be insufficient for learners to acquire confidence with physical examinations [2] or to effectively link findings to diagnosis through clinical reasoning [3].

Simulations that are aligned with sound educational principles and theorems and provide an authentic opportunity for performance practice have been shown to be effective in facilitating the development of clinical competency and satisfaction of learners [4, 5]. Because of this evidence, we have implemented simulation-based educational opportunities for our learners to develop skills in MSK physical examination and clinical reasoning in the orthopaedic clinical clerkship rotation in our university hospital. Role-playing is a novel and effective education method [6] that provides a positive learning experience [7]. Furthermore, role-playing has several advantages, such as saving time and cost in the programme [8], higher performance of students [9], the achievement of decision-making skills [10], and the promotion of reflection and self-efficacy [11]. Nevertheless, few studies have investigated whether practising role-play in educational settings has consequences for clinical practice.

Therefore, the purpose of this study is to assess the effectiveness of a peer role-playing simulation programme to improve the clinical skills of medical students using the mini clinical evaluation exercise (mini-CEX). We developed our study to answer the following question: Do students who experienced peer role-playing as low-fidelity simulation for MSK cases improve their competency in performing a physical examination, exercising clinical reasoning and diagnosing in a clinical setting compared with the control group? Low-fidelity simulation in our study was defined as peer role-plays (student-to-student), and the high-fidelity simulation was defined as other role-plays, for example, with the simulated patient.

## Methods

### Programme competency and assessment

Our simulation programme used peer role-play (student to student) focussing on MSK symptoms. Learners had to use two programme competencies: (1) to perform an MSK physical examination as part of a patient’s first medical visit for the stated problem, ensuring patient safety and comfort; and (2) to present their clinical reasoning process for establishing a clinical diagnosis to their supervisor after the outpatient encounter.

 The study group included 90 fifth-year female medical students who participated as part of their orthopaedic clinical clerkship rotation (our study group consisted only of female students as the study setting was a women’s medical university). All participants had completed the MSK component of coursework in a classroom in the previous academic year, including physical examination, clinical reasoning, and diagnosis. They had been on internal medicine clerkship rotation before the orthopaedic clinical clerkship rotation. Rotations were performed in groups of four or five students.

**Fig. 1:**
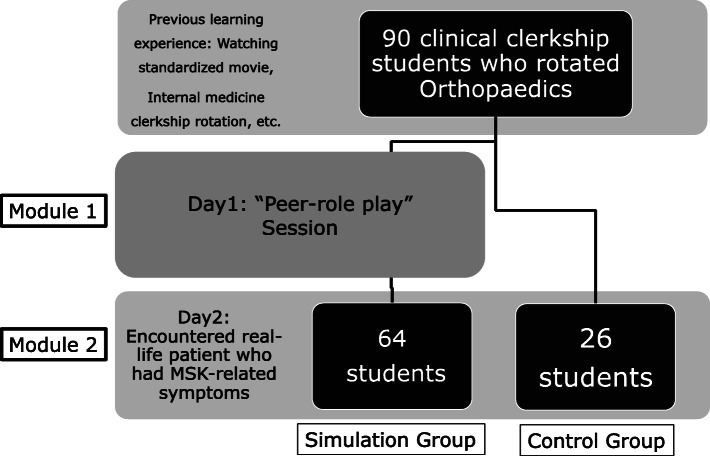
The program structure and the distribution of participants

Students were classified into the simulation group (*N* = 64) and the non-simulation group as the control group (*N* = 26). The simulation group was defined as those who participated in a peer role-play session. The control group was defined as those who, due to schedule circumstances, did not participate in a peer role-play session (Fig. 1).

At the peer role-play session the following day, using a mini-CEX, the students’ supervisor observed them each during an encounter with a first-visit patient at the orthopaedic consultation room, and then rated their performance in different domains. The mini-CEX has been widely applied to assess clinical competencies for medical students and residents in a clinical setting [12, 13]. Several studies have demonstrated that mini-CEX domain scores correlate highly with each other [13–15]. The mini-CEX has proven validity as an assessment of clinical skills through observation.

We compared mini-CEX scores between the simulation group and the control group. The assessment domains in the mini-CEX were: (1) history-taking; (2) physical examination; (3) communication; (4) clinical reasoning and diagnosis; (5) humanity and professionalism; (6) management; and (7) overall clinical competency.

We used a questionnaire to survey participants’ pre-learning status and motivation of prior to introducing the programme as an option. Its purpose was to determine if students might respond well to having such a programme and to get a sense of where their skills and comfort were before the intervention. The content of the questionnaire, which involved the pre-learning status and motivation of participants, was decided through discussions with supervisors and researchers in this study.　The items on the pre-learning status questionnaire comprised (1) experience of watching the standardised educational movie about MSK physical examination before the programme (yes/no), (2) experience of participating in role-playing as an educational opportunity for MSK physical examination during their pre-clinical clerkship rotation (yes/no), and (3) experience of encountering an MSK patient during their previous rotations in other departments (yes/no). The motivation questionnaire points were (4) students’ motivation to improve their skills of MSK physical examination (4-point Likert scale), and (5) students’ motivation to improve their clinical reasoning skills (4-point Likert scale).

### Programme design

The simulation group took part in all modules (Module 1 and Module 2) of the programme on Day 1 and Day 2. The control group took part in only Module 2 on Day 2. The programme consisted of two modules with two sessions in each module.

On Day 1, the first session of Module 1 consisted of ‘peer role-play’ for an MSK case (choice of a spine, upper extremity, or lower extremity case), in which the interaction took place between the simulated patient (a student) and the doctor (another student). The method for the peer-to-peer role-playing session is as follows. First, a student playing the role of a patient decides on a medical complaint to report and creates a simple, 2–3-minute-long scenario for delivering the complaint. This student can create and decide on the content of the scenario freely, without any restrictions on the complaint or the part of the body. The supervisor does not know the content in advance. Then, a student playing the role of a doctor role-plays a medical interview and physical examination for 10 min with the student portraying the patient. The student acting as the doctor summarises their clinical reasoning and diagnosis based on the collected information and findings. They then provide a case report on the role-play to their supervisor. The supervisor evaluates the role-playing session using the mini-CEX and gives feedback to the students. Lastly, the students reflect on the experience and give themselves feedback. The feedback from supervisors and students are delivered verbally and in short texts, respectively.

The second session of Module 1, included the following structural elements to guide learning: recording the interaction to provide feedback; requiring the student to explain their process of the MSK physical examination, clinical reasoning and diagnosis; having the supervisor assist the student, as needed, during the interaction; having the supervisor use the mini-CEX to formally assess the student’s performance; having the supervisor provide a debrief and feedback to the student regarding their performance; and requiring the student to self-reflect to prepare for Module 2.

On Day 2, the first session of Module 2 included the following structural learning elements: First, the students were provided with real-life patient information from a patient’s pre-interview sheet. Next, the student took the patient’s history and performed a physical examination of a patient who had MSK-related symptoms in the consultation room of the orthopaedic surgery department. The supervisor observed and assessed the student’s performance, which had been examined with the real-life patient using mini-CEX. The second session included a debrief of the completed mini-CEX assessment with the student. The physical examination and the clinical reasoning and diagnosis were discussed with the student, and feedback on their oral and written tasks was provided by the supervisor. The student was expected to self-reflect for the next step.

 We confirmed that all participants, including students and real-life patients, provided informed consent. This study was approved by the Tokyo Women’s Medical University Ethics Review Board.

### Data collection and analysis

The mini-CEX scores of the real-life MSK patient encounter in Module 2 were compared between the simulation group (*N* = 64) and the control group (*N* = 26) using the Wilcoxon rank-sum test. The alpha-value is at 0.05. In any event, *p* values less than the alpha-value are, by definition, statistically significant. The answers of the pre-learning status and the motivation to participate prior to the programme as the pre-survey were compared between the simulation group and the control group. The pre-learning status was analysed using Pearson’s chi-square test. The motivation scale of physical examination and the clinical reasoning and diagnosis were analysed using Fisher’s exact test. All analyses were performed using JMP® Pro 15 (SAS Institute Inc., Cary, NC, USA).

## Results

For the role-play group (*N* = 64), we found the following changes in the average mini-CEX scores for medical interview, physical examination, and communication. For history taking, the scores increased from 4.41 at the time of the role-play to 4.64 at the time of the patient encounter. For physical examination, the scores increased from 4.05 at the time of the role-play to 4.27 at the time of the patient encounter. For communication, the scores increased from 4.48 at the time of the role-play to 4.63 at the time of the patient encounter. No statistically significant differences were found. There were cases where the perspectives of clinical reasoning and diagnosis, professionalism, and management could not be assessed during role-play. For that reason, these were omitted when calculating the results.

**Fig. 2:**
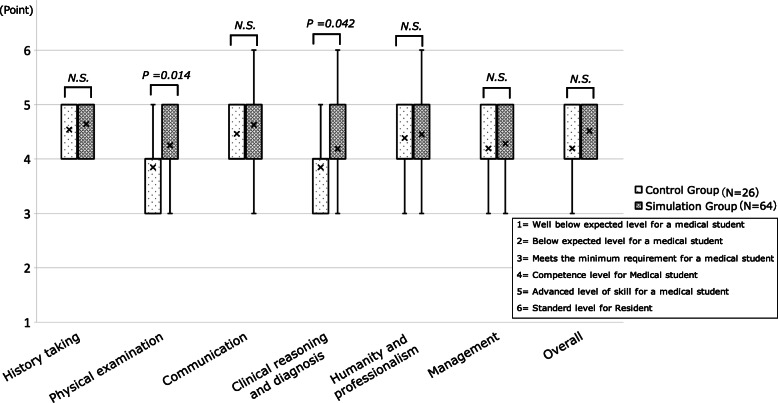
Physical examination and clinical reasoning & diagnosis of mini-CEX score in the simulation group were higher than the control group

The mini-CEX scores of physical examination, clinical reasoning and diagnosis, and overall clinical competency were significantly higher in the simulation group than in the control group (physical examination: *p* < .05, *p* = .014, clinical reasoning: *p* < .05, *p* = .042, overall: *p* < .05, *p* = .016). The history-taking of the mini-CEX scores was significantly different between the simulation and control groups (*p > .*05, *p = .*37). However, communication, humanity and professionalism, and management scores of the mini-CEX assessment were not significantly different between the groups (communication: *p > .*05, *p = .*18, humanity and professionalism: *p > .*05, *p = .*61, management: *p > .*05, *p = .*57) (Fig. 2). These findings suggest that the medical students who took part in the peer role-playing simulation programme had improved their clinical skills of history-taking, physical examination, clinical reasoning and diagnosis, as well as overall clinical competency for the real-life MSK outpatient encounters.

Other results are shown. We analysed the correlation between history-taking, physical examination, and communication in cases with the same complaint (*N* = 13) and cases with different complaints (*N* = 51) during the role-playing and patient encounters to reveal the following. For history-taking, the correlation coefficient was *r* = -.10 for the same complaint and *r* = .57 for different complaints. For physical examination, it was *r* = -.16 for the same complaint and *r* = .27 for different complaints. For communication, it was *r* = .57 for the same complaint and 0.43 for different complaints.

The results of the pre-survey indicate how many students had taken part in the following educational activities regarding MSK physical examination, clinical reasoning and diagnosis before our intervention: 96.9 % of the simulation group and 96.2 % of the control group had watched the standardised educational movie about MSK physical examination; 87.5 % of the simulation group and 88.5 % of the control group had participated in role-playing as an educational opportunity for MSK physical examination during their pre-clinical clerkship rotation, and 17.2 % of the simulation group and 11.5 % of the control group had encountered an MSK patient during one of their previous rotations in other departments (e.g., emergency department, neurology, and rheumatology). There were no significant differences between the simulation group and the control group for watching the educational movie (*p > .*05, *p = .*86), previous role-playing experience (*p > .*05, *p = .*90), encountering an MSK patient (*p > .*05, *p = .*50). Table 1 shows Students’ motivation to improve their skills in the MSK physical examination and the clinical reasoning and diagnosis were not significantly different between the simulation group and the control group (motivation for physical examination: *p > .*05, *p = .*095, motivation for clinical reasoning: *p > .*05, *p = .*079; Table 2). The pre-learning status and motivation were similar between the simulation group and the control group.

**Table 1:** The amount and percentage of students who had each previous learning experience before the rotation of Orthopaedics

	Simulation group (*N* = 64)		Control group (*N* = 26)		
Previous leaning experience	Yes	No	Yes	No	*P value*
Watching educational movie	62 (96.9%)	1 (3.1%)	25 (96.2%)	1 (3.8%)	.86
Role playing as an educational strategy	56 (87.5%)	8 (12.5%)	23 (88.5%)	3 (11.5%)	.90
Encountered an MSK patient	11 (17.2%)	53 (82.8%)	3 (11.5%)	23 (88.5%)	.50

**Table 2:** Students’ motivation to improve their skills of MSK Physical examination and Clinical reasoning

	Simulation group (*N* = 56)					Control group (*N* = 19)					
Program competency	1 (Strongly Disgree)	2 (Disgree)	3 (Neutral)	4 (Agree)	5 (Strongly Agree)	1 (Strongly Disgree)	2 (Disgree)	3 (Neutral)	4 (Agree)	5 (Strongly Agree)	*P value*
Physical examination	0	0	2 (3.57%)	25 (44.6%)	29 (51.8%)	0	0	3 (15.8%)	5 (26.3%)	11 (57.9%)	.095
Clinical reasoning	0	0	3 (5.36%)	26 (46.4%)	27 (48.2%)	0	0	3 (15.8%)	4 (21.1%)	12 (63.2%)	.079

## Discussion

We have provided evidence that simulation-based education is effective in improving students’ skills in performing an MSK physical examination. We used peer role-play as a simulation, with students assuming the role of a patient with MSK symptoms in a clinical clerkship setting. Our findings align with a meta-analysis that reports the effectiveness of simulation-based medical education (SBME) for improving clinical skills among medical learners [16]. Another study shares the effectiveness of SBME in improving diagnostic skills for rheumatoid arthritis and osteoarthritis [17]. Yet another study shows that medical students struggled to improve their MSK physical examination skills during regular clinical clerkship activities. However, small group interactive clinical skills courses with multi-source feedback provided to students produced considerable improvements in students’ clinical skills after several months [5]. These findings underline the importance of clinical skills laboratory sessions, including simulation-based opportunities, in improving clinical skills and reasoning. In our study, we present that short term SBME could be effective in enhancing learners’ skills for MSK physical examination and clinical reasoning during clinical clerkships and that the mini-CEX could be a time-saving and effective workplace-based assessment tool.

Our results indicate that peer role-playing actively improved competency for the MSK physical examination, clinical reasoning and diagnosis in the context of patient and doctor encounters. Our intervention was implemented by facilitating ad-lib encounters based on MSK cases as a practical learning opportunity.

One reason why these findings point toward the fact that peer-to-peer role-playing achieved clinical reasoning competency for real-life patients in a clinical setting is that the study methods are valid and reliable. Regarding SBME, it is worthy to note an article that has reported no significant difference in learning between high- and low-fidelity simulations [18]. This study supports our use of low-fidelity simulation, namely, peer-to-peer role-play, to improve physical examination skills. Our results suggest that peer role-play is a valid form of low-fidelity simulation education. According to one recent review, role-play in health education enhances students’ therapeutic and communication skills. Specific actions that led to associations between scores in the experimental group included practice opportunities and learning through peer-to-peer role-playing. In particular, during the feedback from the supervisor after role-playing, importance was placed on the value of carrying out clinical reasoning and diagnosis based on information gathered from the history-taking and physical examination, and training with that in mind. Importance was also placed on practicing physical examination techniques to aid in clinical reasoning and diagnosis. In the students’ feedback, reflections on the experience included wanting to be able to perform examinations while thinking about clinical reasoning and diagnosis, wanting to be able to accurately and speedily obtain findings from physical examination techniques, wanting to practise more because the experience was difficult, and wanting to keep working hard. As a result, it appears that rises in physical examination and clinical reasoning scores are associated with changes in awareness of the need to perform a physical examination properly to aid clinical reasoning and diagnosis, increases in motivation for the patient encounter the next day, and the timeliness of the simulation.

The advantages of peer-to-peer role-playing include relieving excessive anxiety and allowing students to practise repeatedly, even outside of session hours. Furthermore, role-playing in supervised groups seems to promote reflection and insight not only for students in the patient roles but also for peers observing the group sessions [19].

Accordingly, we found no positive effect or correlation between the role-play and patient encounter scores for cases where the complaint was the same during the role-play and the patient encounter. This could be due to differences in anxiety and context between role-playing and actual outpatient examinations. Even when a student’s role-playing score is low, they may perform favourably the next day, depending on how they reflect on and learn from the role-playing experience immediately after it is over. The opposite may also occur, due to differences in anxiety and context, or when a student neglects to study immediately after the role-playing session. It is possible this can be resolved by improving the accuracy of the scenario or using simulated patients, instead of peer-to-peer role-playing. This could also be considered a limitation of role-playing.

Another study stipulates that the evaluation of the use of role-playing as an educational method by Kirkpatrick’s model showed improved learning outcomes for health profession students [20]. However, there is limited evidence on how this translates to patient outcomes, and no indication of the economic benefit of this type of training compared to other methods [21]. There is a need for future research to consider the optimal format for clinical-based education and its complete role in medical education more fully.

This study suggests that peer role-playing as a low-fidelity simulation and practical learning opportunity will enable the improvement of the competency of medical students in musculoskeletal physical examination as well as clinical reasoning and diagnosis in a clinical setting.

## Limitations

First, since all the subjects in this study were female students (as the study setting was a women’s medical university), it is necessary to conduct a similar study with male students to confirm the generalisability of the results at other medical schools. A second limitation is that the number of participants in each group was different. This is because groups were randomly assigned according to the schedule of the clerkship rotation. Third, the students’ stage of learning development differs slightly from group to group because they rotate through the orthopaedic clerkship over a year. Fourth, it is also possible that the mini-CEX assessment scores may be biased because only one person, a supervisor, assessed all students in the study. The final limitation of this study is that we did not examine whether the advantages of role-playing are retained when the first role-play session was one week or one month prior. There may also be challenges related to maintaining motivation, but supervisors could devise ways to motivate students such as checking on them at intervals. By doing this, it may be possible to retain the benefits of peer-to-peer role-play while increasing opportunities for practice and maintaining or increasing scores. Additional studies are required to confirm the validity of these assessments.

## Conclusions

Concerning practical learning opportunities, peer role-playing as a low-fidelity simulation will enable students to improve the skills of other students regarding MSK physical examination and clinical reasoning and diagnosis in an outpatient encounter during the orthopaedic clerkship. In addition, peer role-playing can be a successful way to practise even when resources are limited due to the Covid-19 pandemic. To establish long-term clinical skills for medical students, the continuous implementation of such simulation-style practices at the workplace will be necessary.

## Data Availability

Data will not be shared publicly to prevent possible inference of the identities and clinical performance of participating students.

## Abbreviations

MSK: Musculoskeletal
mini-CEX: Mini clinical evaluation exercise
SBME: Simulation-based medical education
OSCE: Objective structured clinical examination
